# A Hypercalcemic Crisis Complicating Subcutaneous Fat Necrosis of the Newborn: A Case Report and Literature Review

**DOI:** 10.7759/cureus.65683

**Published:** 2024-07-29

**Authors:** Sara E Marhoon, Ali H Ali, Eman G Elshabrawy

**Affiliations:** 1 College of Medicine, Mansoura University, Mansoura, EGY; 2 Pediatric Nephrology, Mansoura University Children Hospital, Mansoura, EGY

**Keywords:** case report, newborn, deep venous thrombosis, hypercalcemic crisis, subcutaneous fat necrosis

## Abstract

Subcutaneous fat necrosis of the newborn (SFNN) is a rare panniculitis that is characterized by the presence of skin nodules. Although SFNN is a self-limited benign disease, effective follow-up is highly recommended to detect hypercalcemia and other complications early on. A male newborn was admitted twice to the neonatal intensive care unit (NICU). The first NICU admission was for hypoglycemia, and the second was due to late-onset sepsis, in which reddish nodules were detected on the back, flanks, shoulders, and posterior aspects of the legs. At 44 days old, the infant was referred to the emergency department due to a hypercalcemic crisis. Screening for other SFNN complications revealed eosinophilia, hypoglycemia, and nephrocalcinosis. The hospitalization was further complicated by a rare occurrence of deep venous thrombosis. The calcium level was followed up to ensure the patient’s recovery. This case highlights the complications that might follow SFNN and emphasizes the importance of its surveillance.

## Introduction

A hypercalcemic crisis, or malignant hypercalcemia, is characterized by a total serum calcium level above 3.5 mmol/L [1]. This condition represents a rare endocrine emergency that poses a life-threatening risk [1,2]. This risk is particularly significant in pediatrics, as it is even less common than in adults, thus presenting a challenge in identifying its various etiologies such as primary hyperparathyroidism, malignancy, Williams syndrome, hypophosphatasia, glycogen storage disease, idiopathic infantile hypercalcemia, and renal tubular acidosis [1]. An especially unique cause of hypercalcemia in pediatrics is subcutaneous fat necrosis of the newborn (SFNN), a rare panniculitis characterized by the presence of reddish or purplish painful nodules on the back, shoulders, and extremities [3]. The diagnosis of this disease is mainly clinical [3]. Although SFNN is a benign condition, if complicated by hypercalcemia, it can lead to high morbidity and mortality [4]. In this context, we present a rare case of hypercalcemic crisis as a complication of SFNN and the subsequent discovery of other crucial complications.

## Case presentation

A male infant was delivered at term via cesarean section due to fetal macrosomia, with a weight of 5 kg. However, the mother had no history of gestational diabetes mellitus (GDM) or any other medical conditions.

**Figure 1:**
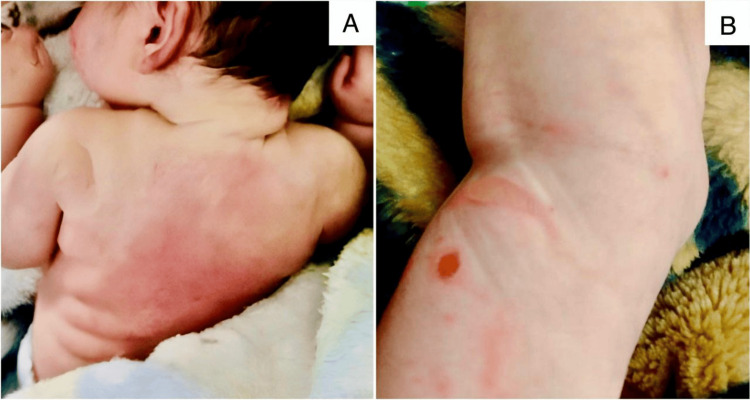
(A) Erythematous, tender plaques on the back and shoulders due to subcutaneous fat necrosis. (B) Red nodules on the posterior aspects of the legs

Following the delivery, mild cyanosis was noted, which necessitated resuscitation measures. The newborn was admitted to the neonatal intensive care unit (NICU) at birth for three days due to unexplained hypoglycemia. One week later, he experienced another NICU admission lasting 21 days due to late-onset sepsis. During this period, he developed erythematous, tender plaques on the back and shoulders, and multiple red nodules on the posterior aspects of the legs (Figure 1). Ultrasound (US) examination of the skin lesions revealed multifocal thickening of the subcutaneous tissue with no evident underlying cystic or solid lesions, thereby supporting the diagnosis of SFNN.

At 44 days old, the infant was referred to our emergency department due to a sudden onset of lethargy, poor activity, and poor feeding, persisting for approximately three days. Upon examination, severe dehydration and dilated veins of the anterior abdominal wall were observed.

Several laboratory investigations were initiated, revealing significant findings, including anemia, leukocytosis, severe hypercalcemia, hypoglycemia, and impaired renal function (Table 1).

**Table 1:** Laboratory investigations

Test	Result	Reference range
Complete blood count		
White blood cell count	18.30 K/μL	4.1-10.9 K/μL
Red blood cell count	3.604 m/μL	4.20-6.30 m/μL
Hemoglobin	11.10 g/dL	12-18 g/dL
Mean corpuscular volume	83.98 fL	80–97 fL
Mean corpuscular hemoglobin	30.80 pg	26-32 pg
Mean corpuscular hemoglobin concentration	36.67 g/dL	31-36 g/dL
Platelets	413.7 K/μL	140-440 K/μL
Kidney function test		
Serum calcium	5.24 mmol/L	2.25-2.87 mmol/L
Serum potassium	4.2 mEq/L	3.5-5 mEq/L
Serum sodium	136 mEq/L	135-145 mEq/L
Serum creatinine	0.9 mg/dL	0.2-0.4 mg/dL
Vitamin assessment		
25-hydroxy vitamin D (total)	41.14 ng/mL	Sufficient: 30-100 ng/mL
Blood glucose test		
Random blood glucose	38 mg/dL	59-126 mg/dL

He was immediately admitted to the pediatric intensive care unit (PICU). Intravenous (IV) fluid administration at 150% of the calculated maintenance fluid requirement, IV furosemide at 1 mg/kg/day after rehydration, oral steroids at 1 mg/kg/day, and amoxicillin-clavulanate were initiated. Other SFNN complications were also assessed through a complete blood count that showed eosinophilia, a renal US that confirmed the presence of bilateral nephrocalcinosis grade III, and a transthoracic echocardiography that showed no significant abnormalities.

Eleven days after PICU admission, the right lower limb began to swell, and the thigh became tender and tense. Duplex US showed echogenic content, suggesting deep venous thrombosis (DVT) in the central femoral vein (CFV), which had previously been catheterized. Consequently, the catheter was promptly removed, and enoxaparin sodium was initiated and continued for two weeks. Additional measures were taken, including the utilization of elastic stockings, elevation of the leg, and ensuring adequate hydration.

**Figure 2:**
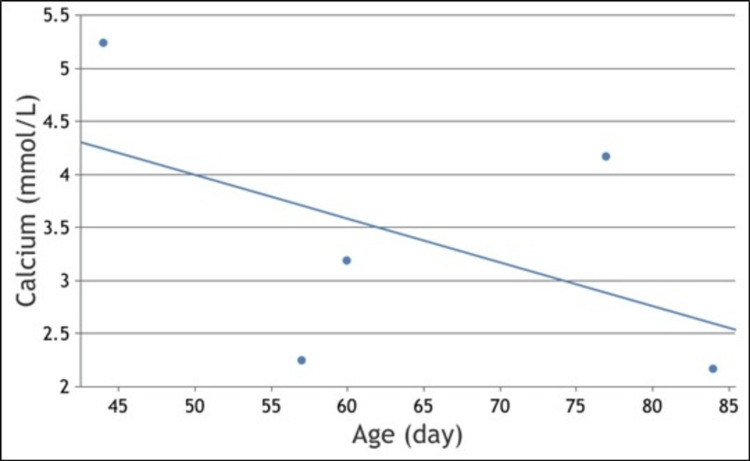
Total calcium level follow-up

Two weeks later, the infant’s general condition was stable. At the time of discharge, the total serum calcium level began to rise, reaching 3.19 mmol/L after being lowered to 2.25 mmol/L during hospitalization. Nevertheless, repeated renal US examinations continued to show the presence of nephrocalcinosis. Following consultation with the endocrinology team, the infant was discharged on alendronate in addition to oral anticoagulants with scheduled outpatient clinic appointments for continued care and follow-up of the calcium levels (Figure 2).

## Discussion

SFNN is a rare panniculitis, mostly found in full-term and post-term infants [5]. The exact pathogenesis of the disease and the associated hypercalcemia are still not fully understood. However, it is suggested that any neonatal distress that might interfere with the blood supply to the fat tissue and cause tissue hypoxia will result in inflammation and subsequent necrosis [6]. This was also noted in our patient, who experienced late-onset sepsis. SFNN has several other risk factors, some for the newborn and others for the mother. Neonatal risk factors include hypothermia, hypoglycemia, anemia, thrombocytosis, perinatal asphyxia, and meconium aspiration [7,8]. This correlates with the patient in our case, who was both hypoglycemic and anemic. Maternal risk factors encompass exposure to cigarettes, cocaine usage during pregnancy, preeclampsia, hypertension, and GDM [7,8].

SFNN is generally a benign and self-limited disease that is usually diagnosed clinically [9]. In some doubtful cases, a skin biopsy may be indicated [4]. However, this condition can be associated with complications such as hypoglycemia, thrombocytopenia, hypertriglyceridemia, eosinophilia, nephrocalcinosis, and, most significantly, hypercalcemia [6,10]. In our case, complications included hypoglycemia, eosinophilia, bilateral grade III nephrocalcinosis, and a hypercalcemic crisis. Nephrocalcinosis persisted despite treatment for hypercalcemia.

In a systematic review of 127 individual cases of SFNN focusing on the association with hypercalcemia [4], 92% of neonates were noted to have skin lesions within the first 28 days of life, and 87% of patients develop hypercalcemia within the first 56 days of life [4]. Seventy-seven percent of cases of hypercalcemia occur within 28 days after the diagnosis of the skin lesions. Hypercalcemia resolves within the first 84 days of life in 93% of patients [4]. Additionally, higher birth weight was substantially associated with a higher risk of developing elevated calcium levels [4], as evidenced in our case. Hypercalcemia can be asymptomatic and tends to become symptomatic when the total blood calcium is at or above 3 or 2 mmol/L of ionized calcium, which is considered moderate hypercalcemia [4,11]. Symptoms of hypercalcemia include irritability, vomiting, poor feeding, hypotonia, failure to thrive, and seizures [4]. In another systematic review that included 206 patients with SFNN, the mean serum calcium concentration was 3.6 mmol/L [12]. Our case had an unexpectedly high calcium level of 5.24 mmol/L, which has not been reported in existing literature before. It is postulated that elevated 1,25-hydroxyvitamin D3, released from the granulomas of the skin lesions, can stimulate the uptake of calcium from the intestine, resulting in hypercalcemia in SFNN [2]. However, the 1,25-hydroxyvitamin D3 level was normal in our patient.

The differential diagnosis for SFNN includes sclerema neonatorum, cold panniculitis, erythema nodosum, cellulitis, hemangioma, histiocytosis, Fabry disease, fibromatosis, and rhabdomyosarcoma. In cases of doubtful clinical presentation, histopathology should be the next step to differentiate between these diseases [4,13].

The first-line treatment options for hypercalcemia include hydration, furosemide after rehydration, IV steroids, IV bisphosphonates, or calcitonin [1]. It is recommended that hypercalcemia screening be conducted for up to six months after the resolution of the skin lesion [4].

Fetal and perinatal thromboses are rare conditions [14]. There have been no reported cases of SFNN complicated by thrombosis in the literature, making our case the first to document this occurrence. The risk factors of thrombosis in this age group include congenital heart disease, perinatal hypoxia, prematurity, polycythemia, respiratory distress syndrome, congenital thrombophilia, surgeries, congenital nephrotic syndrome, congenital metabolic disorders, administration of calcium salts or hypertonic substances, infections, inflammation, hypovolemia, and dehydration. However, the most common risk factor is having vascular catheters, particularly central and umbilical ones [14]. The CFV was catheterized in our patient, which, besides other risks such as dehydration and hypercalcemia, probably contributed to developing DVT.

## Conclusions

The postnatal course might be complicated by unexpected diseases such as SFNN. Although SFNN is considered a benign disease, it has the potential to be hazardous since it may lead to a wide range of complications, especially hypercalcemia, which might cause a hypercalcemic crisis. Regular follow-ups with serum calcium levels over a period of six months are crucial to unveil and address any subsequent complications, thereby facilitating the process of SFNN recovery.
